# Dopamine, behavior, and addiction

**DOI:** 10.1186/s12929-021-00779-7

**Published:** 2021-12-02

**Authors:** Roy A. Wise, Chloe J. Jordan

**Affiliations:** 1 Intramural Research Program, National Institute on Drug Abuse, 250 Mason Lord Drive, Baltimore, MD USA; 2 Behavior Genetics Laboratory, McLean Hospital, Harvard Medical School, 115 Mill Street, Belmont, MA 02478 USA; 3 Division of Alcohol, Drugs and Addiction, Department of Psychiatry, Harvard Medical School, McLean Hospital, 115 Mill Street, Belmont, MA 02478 USA

**Keywords:** Dopamine, Behavior, Addiction

## Abstract

Addictive drugs are habit-forming. Addiction is a learned behavior; repeated exposure to addictive drugs can stamp in learning. Dopamine-depleted or dopamine-deleted animals have only unlearned reflexes; they lack learned seeking and learned avoidance. Burst-firing of dopamine neurons enables learning—long-term potentiation (LTP)—of search and avoidance responses. It sets the stage for learning that occurs between glutamatergic sensory inputs and GABAergic motor-related outputs of the striatum; this learning establishes the ability to search and avoid. Independent of burst-firing, the rate of single-spiking—or “pacemaker firing”—of dopaminergic neurons mediates motivational arousal. Motivational arousal increases during need states and its level determines the responsiveness of the animal to established predictive stimuli. Addictive drugs, while usually not serving as an external stimulus, have varying abilities to activate the dopamine system; the comparative abilities of different addictive drugs to facilitate LTP is something that might be studied in the future.

## Background

Addictive drugs are habit-forming. Here we use the phase “habit-forming broadly [1] to refer to the entire progression toward the stimulus–response end habits [2] discussed in the specialist literature.Rewards are habit-forming because predictive stimuli—reward-predictors as well as punishment-predictors—come to cause dopaminergic burst-firing, and burst-firing enhances or enables the separate development of long-term potentiation (LTP) and long-term depression (LTD) of learned connections between other systems: glutamatergic input pathways and GABAergic output pathways [3]. The primary source of these is in the striatum; the striatum receives sensory inputs from the cortex and sends motor-related outputs that are essential for food-searching and punishment-avoidance. The primary evidence for dopaminergic involvement in reward-driven learning comes from studies of genetically altered mice that cannot synthesize dopamine in the brain. These mice appear normal when born, but they fail to learn food-seeking and, after weaning, die of starvation unless they are force-fed [4]. Such animals have only unconditioned reflexes—“consummatory” reflexes [5]—and, having not learned to feed,—an “appetitive” response [5]—also fail to learn to seek or avoid other rewards. That dopamine is critical for such learning is evident from the dopaminergic recordings of Schultz and colleagues [6, 7] and from recent optogenetic studies that confirm dopaminergic activation as rewarding [8–11].

### Dopamine-deficient animals

Dopamine-deficient animals are born with minimal—learned in utero—knowledge of the environment. These animals have normal reflexes: they can swallow food if it is placed in their mouth [12] and they escape from aversive stimuli [13–15]. They do not, however, learn to search for rewards or avoid aversive stimuli [4, 16]. They must learn to approach the environmental cues that guide behavior, leading to food and other rewards [17, 18], just as they must learn to avoid predictive stimuli that warn of punishers. Learned approach to rewards is an appetitive behavior and is essential for addiction as well as for feeding; indeed, the strongest evidence of drug self-administration involved responses to predictors because the animals, in these cases, rarely have sensory contact with the drug itself [19].

### Dopaminergic burst-firing enables environmental learning

Genetically modified neonates that cannot synthesize dopamine in the brain [4], like adult animals with their dopamine systems lesioned [16] or blocked pharmacologically [20–22]—dopamine challenged animals—fail to find and eat external foods [20, 22] and fail to seek and consume addictive drugs [3, 23–25] or other rewards [21, 26, 27].

The dopamine system is activated by three kinds of external stimuli: rewarding stimuli, punishing stimuli, and novel stimuli. When activated by rewards or punishers, portions of the dopamine system discharge in bursts [6, 28–32], whereas other portions are inhibited [33–35]. Dopaminergic burst discharges involve two or more linked spikes with progressively decreasing amplitude and short inter-discharge intervals (about 60 ms between the first and second discharge and about 120 ms between subsequent discharges) [36]. These discharges cause very local accumulations of dopamine in the striatum, at local peaks of 100 nM or greater concentration, as measured by fast-scan cyclic voltammetry (FSCV) [37].

The primary sensory inputs to the dopamine system—driving this release—are glutamatergic and perhaps cholinergic terminals from cell bodies of the latero-dorsal and pedunculopontine tegmental nuclei [38, 39]; similar burst firing can be activated as well by direct glutamatergic stimulation of dopaminergic neurons in isolated brain slices [40]. In dopamine-intact animals, dopaminergic neurons burst-fire in response not just to rewards or punishers but also to stimuli that reliably precede—and thus predict—rewards and punishers [6, 7, 41].

The burst-firing in response to predictors of rewards or punishers develops with age, as the animal learns about the environment. The burst-responses should not really be seen as *travelling* from the unconditioned rewards and punishers *to* their predictors; rather, the process of burst-firing develops anew in response to predictors that involve a Hebbian mechanism [42, 43]. Hebb has postulated a mechanism by which repeated synaptic input from a (predictor) cell that reliably precedes another (reward) neuron becomes linked to its target. As responses to predictors develop, the burst-responding in response to the actual rewards or punishers is temporarily lost; responsiveness, however—in this case inhibition of firing—appears when the reward or punisher fails to appear at the expected time [44]. When burst-firing develops in response to reward-predictors it enables cellular learning in surrounding synapses; these are glutamate-GABA synapses localized within microns of the sites of dopamine release.

Burst-firing of the dopamine system is only a first step in the learning; the formation of the synapses for searching develops in other cellular elements. Dopamine bursting enables development of long-term potentiation (LTP) and long-term depression (LTD), and, in the striatum, this occurs between glutamatergic sensory inputs and GABAergic motor-related outputs [45, 46]. Dopamine in the striatum reaches and binds to high-affinity D2 dopamine receptors and low-affinity D1 receptors [48, 49]. At high affinity D2 receptors significant binding occurs, making D2 receptors particularly sensitive to phasic decreases in dopamine release. At low affinity D1 receptors less dopamine should be bound, making D1 receptors particularly sensitive to phasic increases in dopamine release. Movements result when D1 receptors are activated and inhibition of movement results when D2 receptors are activated [9]. In behaving animals, activation of D1 and D2 are momentary complements; their activations occur concurrently [50]. Concurrent activation presumably involves activating one subset of muscles (D1) to do something while inhibiting (D2) other sets of muscles, antagonistic muscles, that would normally interfere with the elicited action. The reward-predicting stimuli that lead an animal to anticipate rewards—both natural rewards and drug rewards—are established by this kind of learning [3, 25].

### Dopaminergic pacemaker-firing modulates motivation

Whereas burst responding of the dopamine system is elicited by external stimuli, dopaminergic single discharges also spontaneously occur; these discharges are identified as pacemaker firing because they result from a depolarizing current within the dopaminergic cells themselves [51]. Such discharges can be seen in brain slice preparations even when, in vitro, excitatory inputs have been eliminated [40]. Pacemaker firing is slower than burst firing; it occurs at about half the frequency of burst-firing [51]. The rate of pacemaker firing is modulated by two sources: by increases or decreases of inhibitory inputs from GABA-containing cells [52] and by hormones and peptides that act at receptors on dopaminergic neurons themselves [53–55] or that act through their inputs [55–57]. The tonic modulation of the dopamine system—pacemaker firing, supplemented by episodes of burst-firing—is a correlate of, presumably a cause of, motivational arousal.

Motivational arousal is a state variable; it regulates readiness to respond to external stimuli. While rewards and punishers elicit responses regardless of emotional state, it is predictors of rewards or punishers that depend on motivational arousal. In resting animals, it is pacemaker firing that varies as a function of internal state and determines when, and to what degree, the animal responds to reward-predictors. Burst-firing can also influence motivational arousal; consider the behavior of an animal when a pheromone-emitting conspecific passes nearby. Motivational arousal varies over time and, in resting animals, determines when a previously sated animal starts to become hungry and interested in seeking food.

In a resting animal, the release of dopamine is detected historically by microdialysis [58]. Baseline levels of dopamine are estimated to be around 5 nM [59, 60]; microdialysis can measure dopamine levels this low and much lower; microdialysis—in tetrodotoxin-treated animals—can measure dopamine at 1% of baseline levels [61]. However, microdialysis is an insensitive measure, averaging rather than giving data from single cells; it involves the sampling of extracellular fluids through large (~ 250 microns) push–pull cannulae in the brain, in contact with many dopamine terminals, and it usually gives averages taken over minutes or tens of minutes. One possibility is that basal dopamine levels are near 5 nM at all points throughout the striatum; alternatively, it is possible that microdialysis simply reflects the average of large fluctuations around some unknown actual baseline level. In contrast, however, the alternative—FSCV, for example—measures individual elevations and does not have the sensitivity to detect the low levels of dopamine in resting animals; it is insensitive to dopamine at concentrations below 20 nM [37] and uses “background subtraction” to isolate dopamine fluctuations from noise [62]. FSCV measures peak concentrations that are isolated both in localization and in time. Because the degree of temporal and spatial heterogeneity is not known, it is not clear the degree to which these isolated dopamine peaks contribute to the motivational arousal in active animals. More recently developed techniques involving optical technology, calcium imaging, and genetically-encoded fluorescent protein sensors [63] will give us better methods for assessing pacemaker dopamine discharge.

The evidence implicating a causal role of dopamine in motivational function comes from experiments where the dopamine system has been experimentally manipulated [25]. These include the following: Animals with partial dopamine depletion show reduced energy in learned tasks [64]. Parkinsonian patients with decreased dopamine levels have deficits in speed of hand movements [65] and in willingness to squeeze a dynamometer [66]; when dopamine is replaced by L-DOPA administration, these symptoms decrease [66]. Amphetamine, which augments dopamine release, causes humans to increase effort for monetary rewards [67]. A dopamine uptake inhibitor that doubles baseline dopamine levels increases willingness to work for high-carbohydrate pellets [68]. Restoring dopamine by re-establishing synthesis in dopaminergic neurons restores locomotion and food-seeking in dopamine-deficient mice [69, 70].

The fact that dopamine-depleted animals already have responses to rewards and punishers allows a stronger definition of motivation than has been offered in the past; the level of motivation varies with responsiveness to *predictive stimuli* in the environment. The distinction here is between *predictive* stimuli that lead toward or away from rewards or punishers and *rewarding* and *punishing* stimuli themselves, to which dopamine-depleted animals continue to respond.

Motivation is not a linear function of dopamine levels and may vary with noradrenergic as well as dopaminergic motivation. Motivation is low when dopamine levels are low, and it increases as dopamine levels start to increase. However, when dopamine levels are doubled or tripled—such levels as are induced by self-administered doses of amphetamine [71], cocaine [72], or opiates [73]—motivation is lost [74]. Thus the relation that links dopamine level with motivation appears to be a classic “U”-shaped function; such functions have traditionally been associated with arousal and motivation [75, 76].

### Predictive cues can become aversive

Wheeler and colleagues have suggested conditions in which cocaine-predictive cues can become associated with negative affect [77–79]. The first of these papers discussed “cocaine predictive” cues, but the second paper more correctly characterized them as cues of “delayed cocaine delivery.” The parameters of establishing the association of a sweet-tasting substance with aversive conditioning are of particular interest, in part because people who use addictive drugs sometimes appear to do so *in anticipation of,* or *in fear of,* expected aversive symptoms.

In the Wheeler studies, animals were given series of 30 or 45 min-long, intra-oral taste stimuli that preceded 2 h sessions of intravenous saline or cocaine self-administration. After several days of training the facial expression elicited by the taste cues [77] and the effect of these cues on dopamine release [78] were determined. A taste cue that preceded subsequent saline self-administration caused licking and lateral tongue movements—these are responses driven by sweet solutions—whereas cues that predicted delayed cocaine self-administration had come to cause gagging and gaping—the responses to aversive quinine solutions—[77]. Moreover, the cue predicting saline self-administration increased dopamine release, whereas the cue predicting cocaine self-administration inhibited dopamine release [77]. The critical factor here is that it was the predictor of *delayed* cocaine availability that became aversive. Delayed cocaine availability is not well associated with dopamine release; dopamine release is directly controlled by what happens in seconds after the prediction [44]. The immediate consequence—for the dopamine system—of the cue that predicted delayed cocaine was the absence of dopamine it caused after training with series of 29 or 44 one-minute cue exposures.

### Dopamine and addictive drugs

Roles for dopamine in reward theory [80–82] and a role of reward in addiction [83] were established shortly after dopamine was established as a neurotransmitter. Dopamine was first identified with reward function from anatomical [84] and pharmacological evidence [85–89]; it was subsequently implicated as well in motivational function [90–92]. Dopamine has broadly been associated with the rewarding effects of addictive drugs, particularly in the process of establishing habitual drug intake [24, 93, 94]. However, dopamine plays strongly established roles in the addictive properties of some drugs but is implicated by minimal evidence in others.

*Amphetamine and cocaine* The role of dopamine in the rewarding effects of the psychomotor stimulants—amphetamine and cocaine—are strongly established. Self-administered doses of amphetamine [71] or cocaine [72] elevate dopamine levels over four-fold. Dopamine antagonists at high doses block amphetamine and cocaine self-administration [86, 95, 96]. At low doses the antagonists cause compensatory increases in responding, suggesting that the rewarding effects of the stimulants has been attenuated [86, 95, 96]. Dopamine-selective lesions cause immediate loss of cocaine self-administration when the lesions are complete [97] and temporary loss when they are incomplete [98]. These lesioned animals continue to lever-press for the direct dopamine agonist, apomorphine, following these lesions, confirming that the lesioned animals remember their training history and have normal motor capacity [97, 98]. Finally, cocaine and amphetamine induces long-term synaptic changes in glutamate-GABA synapses in the striatum [99–101].

*Opiates* Heroin self-administration is also clearly dopamine-dependent. It more than triples resting dopamine levels [73], and while the role of dopamine in opiate addiction has been questioned [102], evidence from intravenous heroin self-administration studies makes it clear that animals usually request additional heroin each time their dopamine levels fall below about twice-normal levels [103]. An important possibility in experiments blocking opiate self-administration with dopamine antagonists is that the antagonists act not only at post-synaptic receptors but also at dopamine autoreceptors [104] where they increase dopamine firing and dopamine release. By increasing dopamine release—as heroin alone does not—dopamine antagonists elevate extracellular dopamine at the nerve terminal, desensitizing the system to the antagonist and, in this case, requiring more heroin to be effective. In any case, dopamine antagonists do block opiate self-administration [102]; the lack of compensatory increases in responding for heroin following low doses of dopamine antagonists [102] does not [105] rule out a role for dopamine in opiate reward. Studies of opiate-conditioned place preferences adds to the evidence that opiates are habit-forming—place-preferences address the first element of search-habits, the locomotion to the place where drugs are available—and that their habit-forming effects are blocked by dopamine antagonists [106, 107].

*Nicotine* Self-administration of nicotine also appears to be dopamine-dependent. Nicotine self-administration causes burst-firing of dopaminergic neurons [108, 109] and elevates dopamine levels to 150–200% of baseline [110]. It is disrupted by selective dopaminergic antagonists [111] and selective neurochemical lesions [112]. Nicotine acts at sites and on receptors expressed by dopamine neurons and inhibitory controllers of dopamine neurons, such as local GABAergic cells within the ventral tegmental area (VTA). Deletion of nicotinic receptor subunits, such as β2, abolishes nicotine-induced dopamine release and attenuates nicotine self-administration, and re-expression of β2 restores nicotine’s rewarding effects [113–115]. Nicotine causes conditioned place preferences; this is blocked with dopamine antagonists [116]. Nicotine enables LTP in glutamatergic inputs to the dopamine system and primes the ability of cocaine to induce LTP in the amygdala [117, 118], a structure anatomically related to the striatum [119].

*Ethanol* The evidence that dopamine is important for the rewarding effects of ethanol is also substantial but weaker than that supporting dopamine involvement in stimulant or opiate reward. Part of the problem is that we still have no animal model of self-administration that is sufficient to maintain intoxication [120]; rats can be induced to drink alcohol [121–124], and animals can be made physically dependent on alcohol [125, 126], however, even in already dependent rats, voluntary self-administration that maintains dependence is not seen. Ethanol (and ethanol withdrawal) increases burst-firing in dopaminergic animals [127, 128]; ethanol also increases pacemaker dopaminergic firing [129]. Ethanol can increase dopamine levels to 150–200% of baseline [94], and increases dopamine cell burst-firing as well as pacemaker-like firing in the VTA; note, however, that a subset of VTA dopamine neurons are instead inhibited by ethanol [128] and this might also be important.

Dopamine antagonists decrease lever-pressing for ethanol in a sucrose-fading procedure [130, 131]; this is done in animals that were experienced with ethanol and during intervals of alcohol deprivation. However, the case of alcohol is unusual. In a conditioned place preference study, alcohol is reported to be dopamine-dependent in alcohol-naive animals but not in withdrawn, experienced, animals [132]. One possibility is that a dopamine-independent pathway is also involved in ethanol reinforcement [132, 133]. Ethanol enhances synaptic plasticity in the striatum [101].

*Cannabis* There are many cannabinoids, some of which have psychoactive effects and remain to be studied. The primary psychoactive ingredient in marijuana is ^∆9^-tetrahydrocannabinol (THC). While some studies have reported that this agent is self-administered intravenously by rodents [134] and non-human primates [135] and increases striatal dopamine levels [136–139], other studies suggest that THC is not very rewarding in other animals, such that THC self-administration is modest and difficult to sustain [140, 141]. Newer rodent models of edible or vaporized THC self-administration hold promise [142, 143]. However, species differences in cannabinoid receptor expression and distribution, particularly in the VTA, may underlie differences in the rewarding effects of THC between humans, non-human primates and rodents [144].

THC is an unusual agent; two of its endogenous analogues—anandamide, 2-arachidonylglycerol—are expressed by dopaminergic (and other) neurons and are released when dopaminergic neurons fire; they influence dopamine turnover through actions on inputs to the dopamine system [145, 146]. THC is self-administered into two dopamine-rich regions, the posterior VTA where mesolimbic dopamine cell bodies are found and the posterior ventral striatum, where terminals of that system terminate [147]; these sites of action implicate THC’s actions on the dopamine system in reward function and the use of central drug self-administration suggests that site-specificity is important here.

*Barbiturates and benzodiazepines* Much less is known about self-administered doses of barbiturates or benzodiazepines. Barbiturates [148, 149] and benzodiazepines [150, 151] are self-administered both intravenously and intracranially into the VTA [152, 153] by animals. Benzodiazepines increase VTA dopamine neuron firing and induce LTP in glutamatergic inputs to VTA dopamine neurons through positive modulation of local GABA_A_ receptors [154–157]. At experimenter-selected doses they elevate dopamine levels [158–161] and it has been suggested that they are addictive for this reason [24].

*Caffeine* Caffeine is self-administered by animals [148, 162, 163] and produces conditioned flavor preferences (low doses) or conditioned place aversions (high doses) in rats when injected intraperitoneally or directly into the VTA [164]. A dopamine antagonist injected into the shell of the ventral striatum blocks these place preferences, whereas the antagonist injected into the core of the ventral striatum blocks the conditioned aversive effects [165]. Volatized, inhaled caffeine increases extracellular dopamine levels in the nucleus accumbens shell [166]. The mechanism by which caffeine does so is not clear. The main actions of caffeine are mediated through actions at adenosine receptors that form heteromers with dopamine receptors. However, in human Positron Emission Tomography (PET) studies, caffeine increases D2/D3 receptor availability in the ventral striatum, suggesting caffeine alone does not directly increase dopamine levels in this region [167]. Other studies suggest that caffeine enhances the rewarding effects of other manipulations, such as exercise [168] or ethanol consumption [65, 169].

## Conclusions

Learned behavior—perhaps all or almost-all learned behavior—depends on dopamine function; dopamine deficient animals fail to learn to search for food or other rewards and fail to learn to avoid predictable punishers. Dopamine neurons discharge in bursts when triggered by external stimuli, and this burst-firing enables formation of potentiated glutamate-GABA signaling that is critical for learned searching. Dopamine neurons also discharge in slower single-impulse pacemaker firing and the rate of this firing appears to determine motivation in resting (inanimate) animals. The ability of addictive drugs to cause burst-like discharges in the dopamine system is the broadly assumed correlate of addiction, but the direct evidence for this assumption is linked strongly to amphetamine, cocaine, and opiates; the evidence is weaker for nicotine and alcohol, cannabis, barbiturates, benzodiazepines, and caffeine. The abilities of different addictive drugs to enable long-term potentiation and facilitate habit formation via dopaminergic mechanisms should be compared in future studies.

## Data Availability

Not applicable.
